# Elesclomol-copper therapy improves neurodevelopment in two children with Menkes disease

**DOI:** 10.1172/JCI193107

**Published:** 2025-07-29

**Authors:** Elena Godoy-Molina, Natalia L. Serrano, Aquilina Jiménez-González, Miquel Villaronga, Rosa M. Marqués Pérez-Bryan, Rubén Varela-Fernández, Stephanie Lotz-Esquivel, Alba Hevia Tuñón, Prachi P. Trivedi, Nina Horn, Joseph F. Standing, Víctor Mangas-Sanjuan, Mercè Capdevila, Aurora Mateos, Denis Broun, Svetlana Lutsenko, Ines Medina-Rivera, Rafael Artuch, Cristina Jou, Mònica Roldán, Pedro Arango-Sancho, Mónica Saez-Villafañe, Juan J. Ortiz-de-Urbina, Angela Pieras-López, Marta Duero, Rosa Farré, Jordi Pijuan, Janet Hoenicka, James C. Sacchettini, Michael J. Petris, Vishal M. Gohil, Francesc Palau

**Affiliations:** 1 Complex Chronic Children and Palliative Care Unit, Department of Pediatrics, Hospital Regional Universitario, Málaga, Spain.; 2 Instituto de Investigación Biomédica y Plataforma en Nanomedicina, Málaga, Spain.; 3 Laboratory of Neurogenetics and Molecular Medicine, Center for Genomic Sciences in Medicine, Institut de Recerca Sant Joan de Déu, Barcelona, Spain.; 4 Department of Pediatrics, Complejo Asistencial Universitario de León, León, Spain.; 5 Department of Pharmacy, Hospital Sant Joan de Déu, Barcelona, Spain.; 6 Centro de Atención Temprana Dr. Miguel de Linares Pezzi, Dulce Nombre de Maria Psychopedagogic Institute, Málaga, Spain.; 7 Department of Pharmacy, Complejo Asistencial Universitario de León, León, Spain.; 8 Department of Biochemistry & Biophysics, Texas A&M University, College Station, Texas, USA.; 9 Department of Genetics, Kennedy Centre and Copenhagen University Hospital, Rigshospitalet, Copenhagen, Denmark.; 10 Institute for Child Health, University College London, London, United Kingdom.; 11 Great Ormond Street Hospital for Children, London, United Kingdom.; 12 Department of Pharmacy and Pharmaceutical Technology and Parasitology, University of Valencia, Valencia, Spain.; 13 Interuniversity Research Institute for Molecular Recognition and Technological Development, Valencia, Spain.; 14 Department of Chemistry, Faculty of Sciences, Universitat Autònoma de Barcelona, Cerdanyola de Vallès, Barcelona, Spain.; 15 Menkes International Association, Málaga, Spain.; 16 United Nations (FAO), Rome, Italy.; 17 Department of Physiology, Johns Hopkins University School of Medicine, Baltimore, Maryland, USA.; 18 Neuropsychology Unit, Department of Pediatric Neurology, and; 19 Department of Clinical Biochemistry, Hospital Sant Joan de Déu, Barcelona, Spain.; 20 CIBER for Rare Diseases (CIBERER), ISCIII, Barcelona, Spain.; 21 Department of Pathology,; 22 Confocal Microscopy and Cellular Imaging Unit and; 23 Department of Pediatric Nephrology, Hospital Sant Joan de Déu, Barcelona, Spain.; 24 Departments of Ophthalmology and Biochemistry, University of Missouri, Columbia, Missouri, USA.; 25 Hospital Sant Joan de Déu, Barcelona, Spain.; 26 Division of Pediatrics, University of Barcelona School of Medicine and Health Sciences, Barcelona, Spain.

**Keywords:** Clinical Research, Genetics, Neuroscience, Genetic diseases, Neurodegeneration, Therapeutics

**To the Editor:** Menkes disease (MIM #309400) is a fatal copper (Cu) metabolism disorder caused by pathogenic variants in the X-linked *ATP7A* gene that encodes a Cu transporter. Currently, no FDA-approved treatment for Menkes disease exists, and clinical studies with Cu-histidine (Cu-His) treatment (1) have shown limited efficacy, with outcomes dependent on residual ATP7A activity and the timing of administration (2). Recent preclinical studies have demonstrated that a Cu ionophore, elesclomol-Cu (ES-Cu), significantly improves the Cu-deficiency phenotypes of a mouse model of Menkes disease (3) without inducing toxicity at therapeutic doses (4).

Here, we present clinical and therapeutic responses to ES-Cu in 2 children, NP#1 and NP#2, with Menkes disease (Supplemental Table 1; supplemental material available online with this article; https://doi.org/10.1172/JCI193107DS1). Both patients were diagnosed within the first days of life because of family history. The sequence variants predicted a frameshift in ATP7A mRNA and loss of full-length ATP7A protein, which was confirmed by Western blotting and immunofluorescence staining of ATP7A in patients’ skin fibroblasts (Supplemental Figure 1).

To evaluate the feasibility of treatment with ES-Cu, the Menkes International Association (https://menkesinternational.com) created the Copper(less) Committee. After carefully evaluating the potential risks and benefits, the Committee agreed that both patients were suitable candidates for ES-Cu treatment under an exceptional use clinical protocol authorized by the Spanish Agency of Medicines and Medical Devices.

ES-Cu was first administered to NP#1 at 20 months of age as a weekly subcutaneous injection adjunct to daily doses of Cu-His, which was withheld on days of ES-Cu administration to ensure the daily Cu dose did not exceed the recommended daily allowance. Stage I involved dose escalation of ES-Cu, starting at 4 μg (0.4 μg/kg) and increasing to a maximum of 250 μg once per week. This dose was later adjusted to 125 μg weekly based on tolerability (Supplemental Figure 2A). NP#2 started ES-Cu treatment at 2 months of corrected age. The initial dose for NP#2 was 6 μg, which then escalated to a maximum dose of 140 μg (Supplemental Figure 2B). Both patients exhibited mild-to-moderate skin inflammation around the injection site, which partially responded to topical corticosteroids and oral ibuprofen (Supplemental Figure 2C). Ultrasound imaging revealed fat necrosis at injection sites. Overall, the medication was well-tolerated except for these injection-site side effects.

As an endpoint, we selected neurodevelopmental evolution, using the Bayley Scales of Infant and Toddler Development, Third Edition (Bayley-III). For NP#1, the Bayley-III test was administered every 2 months during the first year of treatment. Before starting ES-Cu, his scores in all domains were below the normal range (<5th percentile), except that for receptive language (Figure 1A). After 2 months of ES-Cu treatment, we observed improvements in all domains, with the most striking improvements in cognitive, expressive language, and fine motor domains, which reached normal percentile ranges by 10 months of treatment, though improvements in the gross motor domain occurred later (Figure 1A). The patient is now 57 months old, walks freely without orthopedic support, is able to run, converses with several back-and-forth exchanges, knows several colors, and can count up to 12.

NP#2 started ES-Cu treatment at 2 months of corrected age when baseline Bayley-III scores of all domains were rated within the normal range (Figure 1B). The goal was to maintain normal ranges during treatment without experiencing delays or loss of developmental milestones associated with Menkes disease. At 29 months corrected age, NP#2 remained within normal ranges across all domains except that for gross motor skills. Lower percentiles in the language domain could be due to a discrepancy in the patient’s primary language (Arabic) and the language used for administering the Bayley-III tests (Spanish).

Comparison of neurodevelopmental status at 20 months of age, at which NP#1 received baseline evaluation before starting ES-Cu treatment, and NP#2, who had received treatment for 18 months, suggests that early initiation of ES-Cu may lead to additional clinical benefits (Figure 1, C–E).

After 3 months of ES-Cu treatment, NP#1 showed pili torti, trichorrhexis nodosa, and a lack of melanin granules (Supplemental Figure 3, A–C). Notably, after 19 months of treatment, pili torti were reduced, and melanin granules were observed in the hair structure (Supplemental Figure 3, D–F). Similarly, at baseline and 16 months of corrected age, NP#2’s hair did not contain melanin granules, but they appeared at 22 months of corrected age (Supplemental Figure 3, G–I). 3D structural analysis and autofluorescence imaging by confocal microscopy in NP#1 demonstrated improvements in hair structure (Supplemental Figure 3, J–M).

Overall, blood Cu and ceruloplasmin levels remained relatively stable in the lower normal range for both patients (data are shown for NP#1 in Supplemental Figure 3N). For NP#1, dopamine remained normal, and norepinephrine and epinephrine remained low (Supplemental Figure 3O). Additionally, Cu and biogenic amines related to catecholamine metabolism, such as HVA/MHPG (homovanillic acid/3-methoxy-4-hydroxyphenylglycol), were measured in CSF of NP#1 at weeks 17 and 69 (Supplemental Table 1). Cu was detected at both time points (5 μg/L at week 17 and 2.1 μg/L at week 69; normal range: 4.2–19 μg/L), and HVA/MHPG ratios were 13 and 16.8 nmol/L at each time point, respectively (normal range: 5–30 nmol/L).

Our findings suggest that ES-Cu has therapeutic benefits on various tissues, particularly the brain, implying that ES-Cu can facilitate Cu delivery across the blood-brain barrier. However, we found that connective tissue defects persist, resulting in lung disease, brain vascular tortuosity, and bladder diverticula. These are likely related to insufficient metallation of lysyl oxidases, which cross-link collagen and elastin. In summary, our results with ES-Cu, combined with Cu-His, provide preliminary evidence of the safety and efficacy of this promising treatment regimen for Menkes disease; however, a future clinical trial with additional patients is required to fully assess the therapeutic benefits.

For case reports and detailed methods, including information regarding sex as a biological variable, statistics, study approval, data availability, author contributions, acknowledgments, and funding, see the Supplemental Methods.

## Funding support

This work has been funded by the Menkes International Association (MIa), the Amigos de Nono Foundation, the Ramón Areces Foundation, award CIVP18A3913, and the Spanish Research Agency (AEI) award PID2020-114655RB-I00 to FP. This work is the result of NIH funding, in whole or in part, and is subject to the NIH Public Access Policy. Through acceptance of this federal funding, the NIH has been given a right to make the work publicly available in PubMed Central.

• National Institute of General Medical Sciences, awards R01GM143630 and R35GM152102 to VMG.

• National Institute of Diabetes and Digestive and Kidney Diseases, award R01DK131190 to MJP.

## Supplementary Material

Supplemental data

Unedited blot and gel images

## Figures and Tables

**Figure 1:**
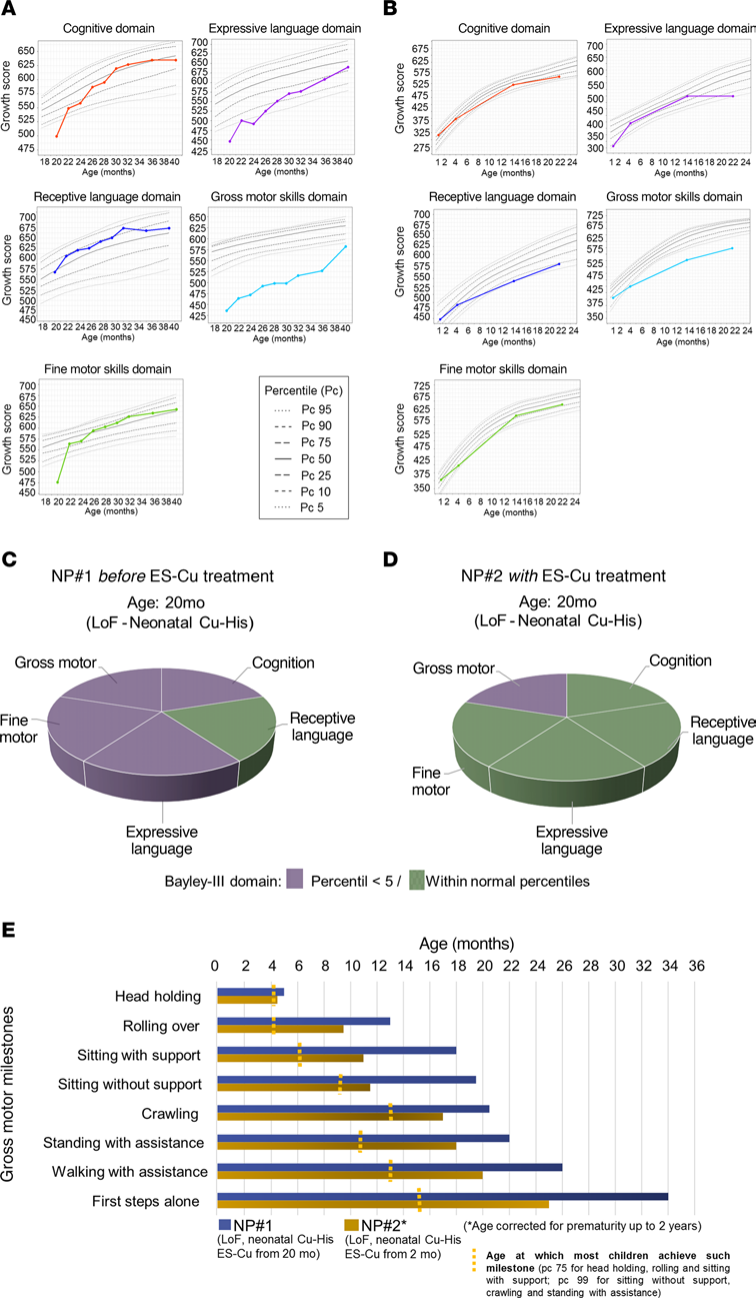
Evolution of neurodevelopmental milestones in NP#1 and NP#2 following the treatment with ES-Cu. (**A** and **B**) Measurements for each neurological domain were taken using the Bayley-III scale. (**A**) For NP#1, evaluations occurred every 2 months during the first 20 months (20–40 months of age), while (**B**) NP#2 was assessed at 4 intervals. (**C** and **D**) Comparison of Bayley-III domain scores at age 20 months between (**C**) NP#1 and (**D**) NP#2. (**E**) Comparison of gross motor milestones acquisition between NP#1 and NP#2. LoF, loss of function.

## Version 1 07/29/2025

In-Press Preview

## Version 2 10/01/2025

Electronic publication

## References

[1] Sarkar B (1993). Copper-histidine therapy for Menkes disease. J Pediatr.

[2] Kaler SG (2008). Neonatal diagnosis and treatment of Menkes disease. N Engl J Med.

[3] Guthrie LM (2020). Elesclomol alleviates Menkes pathology and mortality by escorting Cu to cuproenzymes in mice. Science.

[4] Zulkifli M (2025). Elesclomol rescues mitochondrial copper deficiency in disease models without triggering cuproptosis. J Pharmacol Exp Ther.

