# Supplementary material for: Elesclomol-copper therapy improves neurodevelopment in two children with Menkes disease
Source: J Clin Invest. 2025 Jul 29;135(19):e193107. doi: 10.1172/JCI193107 (PMC12483558; doi:10.1172/JCI193107)
Supplement: Supplemental data [file jci-135-193107-s099.pdf]

Elesclomol-copper therapy improves neurodevelopment in two children with Menkes disease

Supplemental Material

Table of Contents

Authors and Affiliations
Case reports
Methods
ES-Cu formulation
Genetic and protein studies
Confocal microscopy imaging for hair analysis
Statistical analysis
Study approval
Data availability
Author contributions
Acknowledgments
Supplemental Tables
Table S1. Molecular variants, and baseline clinical and biochemical findings of patients before treatment with ES-Cu. Cerebrospinal fluid metabolic parameters in NP#1
Supplemental Figures
Figure S1. Genetic and molecular characterization of ATP7A in NP#1 and NP#2
Figure S2. Dose escalation and skin reaction
Figure S3. Hair and biomarker analysis and monitoring

Elena Godoy-Molina[1,2,*], Natalia L. Serrano[3*,#], Aquilina Jiménez-González[4], Miquel Villaronga[5], Rosa M. Marqués Pérez-Bryan[6], Rubén Varela-Fernández[7], Stephanie Lotz-Esquivel[3], Alba Hevia-Tuñón[4], Prachi P. Trivedi[8], Nina Horn[9], Joseph F. Standing[10,11], Víctor Mangas-Sanjuan[12,13], Mercè Capdevila[14], Aurora Mateos[15,16], Denis Broun[15], Svetlana Lutsenko[17], Ines Medina-Rivera[18], Rafael Artuch[19,20], Cristina Jou[21], Mònica Roldán[22], Pedro Arango-Sancho[23], Mónica Saez-Villafañe[7], Juan J. Ortiz-de-Urbina[7], Angela Pieras-López[5], Marta Duero[5], Rosa Farré[5], Jordi Pijuan[3,20], Janet Hoenicka[3,20], James C. Sacchettini[8], Michael J. Petris[24], Vishal M. Gohil[8] & Francesc Palau[3,20,25,26$]

[1] Complex Chronic Children and Palliative Care Unit, Department of Pediatrics, Hospital Regional Universitario, Málaga, Spain.
[2] Instituto de Investigación Biomédica y Plataforma en Nanomedicina (IBIMA Plataforma BIONAND), Málaga, Spain.
[3] Laboratory of Neurogenetics and Molecular Medicine, Center for Genomic Sciences in Medicine, Institut de Recerca Sant Joan de Déu, Barcelona, Spain.
[4] Department of Pediatrics, Complejo Asistencial Universitario de León, León, Spain.
[5] Department of Pharmacy, Hospital Sant Joan de Déu, Barcelona, Spain.
[6] Centro de Atención Temprana Dr. Miguel de Linares Pezzi, Dulce Nombre de Maria Psychopedagogic Institute, Málaga, Spain.
[7] Department of Pharmacy, Complejo Asistencial Universitario de León, León, Spain.
[8] Department of Biochemistry & Biophysics, Texas A&M University, College Station, TX, USA.
[9] Department of Genetics, The Kennedy Centre and Copenhagen University Hospital, Rigshospital, Copenhagen, Denmark.
[10] Institute for Child Health, University College London, UK.
[11] Great Ormond Street Hospital for Children, London, UK.
[12] Department of Pharmacy and Pharmaceutical Technology and Parasitology, University of Valencia. Valencia, Spain.
[13] Interuniversity Research Institute for Molecular Recognition and Technological Development, Valencia, Spain.
[14] Department of Chemistry, Faculty of Sciences, Universitat Autònoma de Barcelona, Cerdanyola de Vallès, Barcelona, Spain.
[15] Menkes International Association, Málaga, Spain.
[16] United Nations (FAO), Rome, Italy.
[17] Department of Physiology, Johns Hopkins University School of Medicine, Baltimore, MD, USA.
[18] Neuropsychology Unit, Department of Pediatric Neurology, Hospital Sant Joan de Déu, Barcelona, Spain.
[19] Department of Clinical Biochemistry, Hospital Sant Joan de Déu, Barcelona, Spain.
[20] CIBER for Rare Diseases (CIBERER), ISCIII, Barcelona, Spain.
[21] Department of Pathology, Hospital Sant Joan de Déu, Barcelona, Spain.
[22] Confocal Microscopy and Cellular Imaging Unit, Hospital Sant Joan de Déu, Barcelona, Spain.
[23] Department of Pediatric Nephrology, Hospital Sant Joan de Déu, Barcelona, Spain.
[24] Departments of Ophthalmology and Biochemistry, University of Missouri, Columbia, MO, USA.
[25] SJD Únicas Center, Hospital Sant Joan de Déu, Barcelona, Spain.
[26] Division of Pediatrics, University of Barcelona School of Medicine and Health Sciences, Barcelona, Spain.

* Contributed equally
[#] Current address: Engrail Therapeutics, Inc.
[$] Corresponding author: Prof. Dr. Francesc Palau, Institut de Recerca Sant Joan de Déu & CIBERER
C/ Santa Rosa 39-57, 08950 Esplugues de Llobregat, Spain. +34-610188076. francesc.palau@sjd.es

CASE REPORTS

Named patient 1 (NP#1) is a full-term newborn male who presented with generalized hypotonia, kinky hair, and hypothermia. NP#1 had a previous biological half-sibling who was diagnosed at seven months of age with severe seizures. He was a carrier of a pathogenic variant and received treatment with Cu-His but died at nine months of age (1). NP#1 diagnosis was confirmed at 4 days of age by detecting the pathogenic *ATP7A* variant, c.3556delG (p.Glu1186Ser*fs*Ter3). Blood plasma levels of copper and ceruloplasmin were low compared to the newborn normal range (Cu <320 µg/L [400-1400 µg/L] and ceruloplasmin <95 µg/L [106-179 µg/L]). Starting on day five, NP#1 received daily subcutaneous Cu-His at a dose of 250 µg/12 hours, which was reduced to 250 µg/day after the age of 1 year. Despite early Cu-His treatment, the patient showed a marked delay in reaching neurodevelopmental milestones. At 20 months of age, before starting ES-Cu treatment, the patient had hypotonic posture, myopathic facies, pale and lax skin, kinky hair, joint instability, kyphosis, pectus carinatum, and oropharyngeal dysphagia with impaired efficacy. Chronic lung disease with tachypnea and mild subcostal retractions were consistent with the emphysematous bullae seen on a previous computed tomography (CT) scan performed at 19 months of age. A bladder diverticulum had been diagnosed by ultrasound. Neurological examination evidenced mild bilateral ptosis, preserved oculomotor movements, and central hypotonia. In the supine position, his lower limbs rested in an open-book position. He had intentional vocalization without clearly expressing words except “no” and “mama.” He had good social interaction. Tendon reflexes were present and symmetrical without tremors. There was no history of seizures, and the baseline electroencephalogram (EEG) was normal. A brain magnetic resonance image (MRI) performed at 14 months revealed tortuosity of intracranial arteries, basilar dolichoectasia, slight delay in the myelination pattern, and slight supratentorial atrophy. Biochemical studies revealed normal blood copper and ceruloplasmin levels, normal plasma dopamine and epinephrine, low norepinephrine, normal urea and creatinine, and slightly elevated

levels of cystatin C and urinary ß$_2$-microglobulin without other signs of tubulopathy (Supplemental Table 1).

NP#2 is a preterm newborn (30 weeks and 5 days of pregnancy) from a monozygotic twin pregnancy who was prenatally diagnosed with Menkes disease due to the prior diagnosis of the same condition in an older sibling, all carrying the c.2626+1G>A pathogenic variant in *ATP7A*. The older brother passed away at 26 months from severe neurological impairment. NP#2 and his identical twin started Cu-His treatment (250 µg/12hr) at 36 hours of life. At 2 months of chronological age (equivalent to 1 week corrected age), both were admitted for severe respiratory distress and hypoxemia, requiring intubation and assisted ventilation. The twin brother faced irreversible deterioration and died shortly after admission. In contrast, NP#2 improved and was discharged home after 28 days in the hospital. Before initiation of ES-Cu treatment, NP#2 was 2 months of age (corrected for prematurity). At baseline evaluation, the patient was in good general condition, with skin hypopigmentation and hypopigmented brittle hair, a thin appearance with little fatty tissue and pectus excavatum. The cardiac and pulmonary evaluation was normal, and he had mild axial hypotonia and good sucking reflex. Biochemical studies revealed normal blood copper and ceruloplasmin levels, normal urea and creatinine, and slightly elevated blood cystatin C and urinary ß$_2$-microglobulin (Supplemental Table 1).

METHODS

Sex as a biological variable

Our study exclusively examined male individuals.
Menkes disease is an X-linked recessive disorder that affects males; only a few females have been reported.

To provide scientific support and evaluate the feasibility of possible treatment and dose regimen with ES-Cu (2, 3), the Menkes International Association (MIa, https://menkesinternational.com) created the Copper(less) Committee, an ad hoc international multidisciplinary expert board. After

careful consideration of potential risks and benefits, the Committee agreed that NP#1 was a suitable candidate for ES-Cu treatment under an exceptional-access investigational clinical protocol authorized by the Spanish Agency of Medicines and Medical Devices (AEMPS) and SJD Children's Hospital Ethics Committee. For NP#2, the Ethics Committee of the León University Hospital approved the exceptional treatment with the support of the AEMPS. Informed consent was obtained from both children's parents. The Copper(less) Committee used the Bayley Scales of Infant and Toddler Development, Third Edition (Bayley-III) for neurodevelopmental assessment. In addition, a systematic protocol included hair morphology and blood biomarkers for follow-up.

ES-Cu formulation

CIPLA Ltd. synthesized and donated ES-Cu to MIa, formulated for use by the Pharmacy Departments of SJD Children's Hospital and Leon University Hospital. The Copper(less) Committee recommended a dose based on conservative allometric scaling from nonclinical studies in *mo-br* mice and the human equivalent dose (HED) (4), as well as the recommended dietary allowance (RDA) of Cu for pediatric patients (5). The treatment plan followed an n-of-1 protocol, with the patient's baseline condition as an internal control. The Committee proposed an initial weekly dose of 4 µg (0.4 µg/kg) of ES-Cu (calculated in terms of Cu) with a progressive increase until reaching a weekly dose of a maximum dose of 250µg .

Composition for 10 mL:

1. Elesclomol-Cu^{2+} complex……………………….……10 mg
2. Sulfobutylether-β-cyclodextrin ……………………….. 2 g
3. Sterile Water for injection qs………………………..…10 mL
   [NaOH 0.05N adjust pH to 7.2 – 7.6]

Method of preparation protocol (clear orange solution after sterile filtration):

- Prepare a 20% sulfobutylether-β-cyclodextrin (Captisol®) solution. In a syringe, dissolve sulfobutylether-β-cyclodextrin with sterile water.
- Weigh the necessary quantity of elesclomol-Cu^{2+} complex.

- In a sterilized 50 mL glass container, place the ES-Cu powder and moisten the powder drop by drop with the sulfobutylethe-β-cyclodextrin solution, otherwise, it is very difficult for the cyclodextrin to incorporate the complex (this is the most delicate step), in sufficient quantity to obtain the maximum copper richness. Initially, it is a very cloudy suspension.
- Perform the sonication process at 45ºC for 1 hour to achieve maximum solubility, alternating with 30 minutes of orbital stirring at 250 rpm. Repeat this process 5 times (total 7.5 hours). Maintain orbital stirring all night.
- Next day, measure pH (initially pH 4) and adjust with 0.05 N NaOH solution to achieve pH 7.2-7.6.
- Take approximately 0.5 mL of the sample and filter through 0.22 µm to determine the concentration of Cu^{2+} in the solution (Laboratory).
- Considering the concentration obtained in the previous step, in the laminar flow cabinet prepare the vials by filtering through a 0.22 µm sterilizing filter with the necessary volume for the individualized doses of elesclomol-Cu^{2+}.
- Concentration of Cu^{2+} in the solution is determined by ultra-performance liquid chromatography (UPLC).
- Label the vials.

Conservation and stability:

- Pack in sterile amber glass vials.
- Store at 2-8 ºC or room temperature.
- Stability for 90 days.

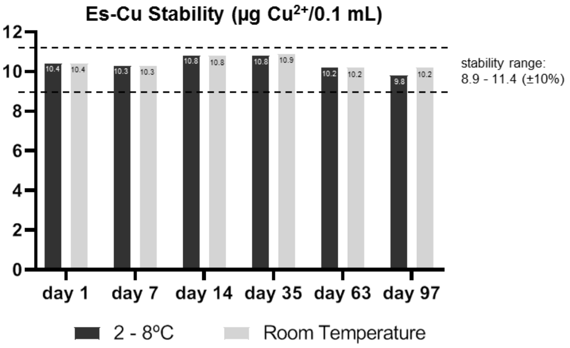

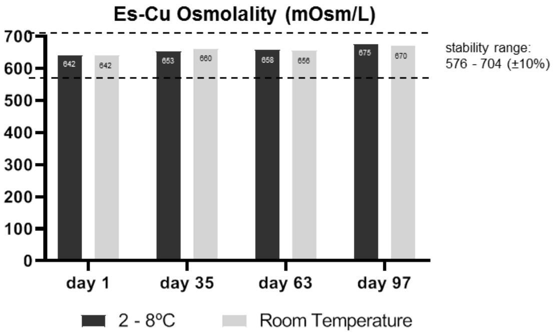

ES-Cu dose calculations

Scaling of ES-Cu dose from mice to humans to set total rescue cumulative dose:

It is unclear whether scaling should follow allometric principles in that if copper from ES-Cu is cleared, and because smaller organisms have higher per-weight clearance, a lower per kilogram dose should be used, or whether copper delivered from ES-Cu remains in the body. Therefore, replacement should be on an equivalent per kg basis to that used in mice.

A 7.25 mg/kg dose was used in 4 g neonatal mice in Guthrie et al. (2020). The mouse dose was therefore:

7.25 mg/kg x 0.004 kg = 0.029 mg

Dose calculation using allometric scaling for a 9.5 kg child:

Using a 2/3 exponent (most conservative scenario):

$$0.029 \text{ mg} \times (\frac{9.5 \text{ kg}}{0.004 \text{ kg}})^{0.67} = 5.30 \text{ mg}$$

Using a 3/4 exponent:

$$0.029 \text{ mg} \times (\frac{9.5 \text{ kg}}{0.004 \text{ kg}})^{0.75} = 9.87 \text{ mg}$$

Dose calculation using linear scaling for a 9.5 kg child:

$$0.029 \text{ mg} \times (\frac{9.5 \text{ kg}}{0.004 \text{ kg}})^{1} = 68.88 \text{ mg}$$

Since the fraction of Cu in ES-Cu is reported to be 3.98 / 29 = 0.137 in Guthrie *et al*. (2020), this relates to rescue Cu doses of 0.727, 1.35, and 9.45 mg of Cu, respectively. As these rescue Cu doses exceed the maximum safety daily Cu dose, we decided to achieve the rescue dose in several weekly doses to get to a target in terms of cumulative dose.

Dose protocol

The Committee established the treatment plan following an n-of-1 treatment protocol, with the patient's baseline condition serving as an internal control. To avoid copper deprivation and due to ethical reasons, we decided to maintain Cu-His to ensure stable levels of serum copper, so our main challenge was to find a balance between an escalating dose of ES-Cu and avoid potential toxicity that could arise from the addition of both forms of copper administration (ES-Cu and Cu-His). Therefore, we decided to omit Cu-His treatment on the day ES-Cu was administered, continuing the other six days of the week at a fixed dose of 250 µg/day. Serum copper and ceruloplasmin levels were measured weekly to ensure circulating copper remained within the normal range.

Genetic and protein studies

Fibroblasts culture. The NP#1's fibroblasts were kindly provided by Dr. D. Martinelli from the Ospedale Pediatrico Bambino Gesù, Rome, the NP#2 fibroblasts were cultured from skin biopsy obtained at Leon University Hospital, and healthy control fibroblasts were provided by Sant Joan de Déu Children's Hospital Biobank. Fibroblasts were cultured in DMEM high-glucose (Sigma-Aldrich) supplemented with 10% (v/v) fetal bovine serum (FBS; Sigma-Aldrich), 2 mM L-glutamine (Sigma-Aldrich) and 100 mg/ml penicillin-streptomycin (Sigma-Aldrich) at 37°C in a 5% CO_2 incubator.

RNA isolation and real-time quantitative PCR analysis. Total RNA was extracted from fibroblasts using the Direct-zol RNA miniprep kit (Zymo Research) according to the

manufacturer's protocol. Maxima™ First Strand cDNA synthesis for RT-qPCR (Thermo Fisher) was used for the reverse-transcriptase (RT) reaction, and all real-time quantitative RT-PCR reactions were carried out three times in triplicate on a QuantStudio 6 Real-Time PCR System (Applied Biosystems) using FastStart Universal SYBR Green Master Rox (Roche). Amplification was performed using 50 ng of cDNA per reaction at 50ºC for 2 minutes, 95°C for 10 minutes, followed by 45 cycles at 95°C for 15 seconds, 60°C for 1 minute, and 72°C for 30 seconds. *ATP7A* gene expression (forward:5'-GCTACCTTGTCAGACACGAATGAG-3' and reverse:5'-TCTTGAACTGGTGTCATCCCTTT-3') was quantified by the standard curve method and samples were normalized to glyceraldehyde-3-phospathe dehydrogenase (*GAPDH*) (forward:5'-AGCGAGATCCCTCCAAAATC-3' and reverse:5'-AATGAGCCCCAGCCTTCTC-3') and phosphoglycerate kinase-1 (*PGK1*) (forward:5'-TTCATGTGGAGGAAGAAGGGAA-3' and reverse:5'-ACATAGACATCCCCTAGCTTGG-3') housekeeping genes.

Western blot analysis. Cells were lysed in a solution containing 50 mM Tris HCl pH 7.4, 1.5 mM $MgCl_2$, 5 mM EDTA, 1% Triton X-100, 50 mM NaF, and 1 mM Na_2VO_3, and protease inhibitor cocktail (Complete Mini-Protease Inhibitor Cocktail, Roche), protein concentration was determined by BCA method (Thermo Fisher Scientific). 50 µg of protein was resolved on 4-15% gradient bis-acrylamide gels (Bio-Rad) and transferred onto PVDF membranes (GE Healthcare). The membranes were blocked with 5% defatted milk in TBS-0.1% Tween 20 buffer (25 mM Tris, 50 mM NaCl, 2.5 mM KCl, 0.1% Tween-20). Afterward, the membranes were blotted with the specific primary antibodies α-ATP7A (1:500; SC-376467, Santa Cruz) and α-TUBULIN (1:8000; T6199, Sigma-Aldrich), which were detected using secondary antibodies coupled to horseradish peroxidase. The chemiluminescence signal was visualized by iBright™ CL1000 Imaging System (Thermo Fisher Scientific).

Immunofluorescence. Fibroblasts were seeded onto glass coverslips and fixed in 4% paraformaldehyde (PFA) for 20 minutes. Cells were permeabilized with 0.2% Triton in phosphate

buffered saline (PBS) for 30 minutes and blocked with 1% bovine serum albumin and 4% serum in PBS. The primary antibody α-ATP7A (1:100; SC-376467, Santa Cruz) was incubated overnight at 4°C, and the Alexa Fluor® 488-labeled secondary conjugated antibody (1:500; A11029, Thermo Fisher) was incubated for 2 hours. Coverslips were mounted with Fluoromont-G with DAPI (ThermoFisher, 00-4959-52). Images were acquired with a Leica DMI3000B fluorescence microscope (Leica Microsystems).

Confocal microscopy imaging for hair analysis

Confocal microscopy analysis was performed by Leica TCS SP8, equipped with a white light laser and Hybrid spectral detectors (Leica Microsystems GmbH). All experiments employed an x10 (0.75 NA) an x20 (0.75 NA) dry objectives. Hair autofluorescence was excited with an argon laser (488 nm) and a white light laser (561 nm), with detection in the ranges of 500-550 nm and 610-795 nm, respectively. The reflection image (grey channel, excited at 488 nm with emission at 480 to 490 nm) visualized the hair medulla. Optimized emission detection bandwidths were configured to prevent inter-channel crosstalk, and a multitrack sequential acquisition setting was utilized. The confocal pinhole was set to 1 Airy unit, and z-stack acquisition intervals were chosen to satisfy Nyquist sampling criteria. Maximum intensity projections were generated using LAS X software (version 3.1.5).

Statistical analysis

All data are expressed as mean ± standard deviation (SD). The specific test applied in each case is indicated in the figure legends. Statistical analysis were performed using the GraphPad Prism software (version 8.0.1).

Study approval

The study was approved by the Healthcare Ethics Committee and the Clinical Research Ethics Committee of Sant Joan de Déu Children's Hospital and Research Institute (ART-06-23 number and clinical protocol study A-GEN-PC-0002-01) and the Ethics Committee of Leon University Hospital. It was conducted in accordance with the ethical principles stated in the Declaration of Helsinki. In both cases, the parents gave informed consent.

Data availability

Data on the findings of this study are included in the main article and supplementary material. Personally identifiable patient values are not given due to ethical reasons. Further information is available from the corresponding author upon request.

Author contributions

EG-M, NLS, and FP initiated and jointly supervised the study. EG-M, NLS, AJ-G, AH-T supervised clinical management. MV, RV-F, MS-V, JJOZ, AP-L, MD, and RF performed and supervised ES-Cu formulation in the two hospitals. RMMP-B and IM-R performed and analyzed Bayley-III scale. EG-M, NLS, SL-E, PPT, NH, JFS, VM-S, MC, AM, DB, SL, JH, JCS, MJP, VMG, and FP are the members of the Copper(less) Committee. RA, CJ, MR, and PA-S performed and interpreted blood, urine, CSF, and hair studies at SJD Children's Hospital. JP and JH performed cellular and molecular experiments in Supplemental Figure 1. FP, NH, and VMG wrote the first draft of the manuscript. All authors revised the manuscript and decided to publish the paper.

Acknowledgements and funding

We are grateful to the parents for their participation and support. Dr. Y. Hamied, CEO of CIPLA, who donated the ES-Cu to undertake the study, and Dr. D. Martinelli, who kindly provided

NP#1's fibroblasts. We are thankful to Anna Aguilar-Ros and Nazareno Lascano for their collaboration. This study has been financed by the Menkes International Association (MIa), the Amigos de Nono Foundation, and the Ramón Areces Foundation grant CIVP18A3913. FP was also supported by the Spanish Research Agency (AEI) grant PID2020-114655RB-I00. VMG was supported by the National Institute of General Medical Sciences awards R01GM143630 and R35GM152102. MJP was supported by the National Institute of Diabetes and Digestive and Kidney Diseases award R01DK131190. JP is the recipient of a postdoctoral contract from CIBERER.

Conflict of interest

EG-M serves as a medical advisor to Engrail Therapeutics, Inc. NLS currently holds the position of Medical Director at Engrail Therapeutics, Inc. VMG and MJP are consultants to Engrail Therapeutics, Inc. FP acts as a scientific and medical advisor to Engrail Therapeutics, Inc.

SUPPLEMENTAL REFERENCES

1. Gerdes AA, Møller LB, Horn N. Ethics in pre-ART genetics: a missed X-linked Menkes disease case. *J Assist Reprod Genet.* 2023;40(4):811-816.
2. Gohil VM. Repurposing elesclomol, an investigational drug for the treatment of copper metabolism disorders. *Expert Opin Investig Drugs* 2021; 30(1):1–4.
3. Nagai M, Vo NH, Ogawa LS, et al. The oncology drug elesclomol selectively transports copper to the mitochondria to induce oxidative stress in cancer cells. *Free Radic Biol Med*. 2012; 52(10):2142–50.
4. Nair A, Jacob S. A simple practice guide for dose conversion between animals and humans. *J Basic Clin Pharm* .2016;7(2):27.

5. Trumbo P, Yates AA, Schlicker S, Poos M. Dietary reference intakes: vitamin A, vitamin K, arsenic, boron, chromium, copper, iodine, iron, manganese, molybdenum, nickel, silicon, vanadium, and zinc. *J Am Diet Assoc.* 2001;101(3):294–301.

Supplemental Table 1. Molecular variants, and baseline clinical and biochemical findings of patients before treatment with ES-Cu. Cerebrospinal fluid metabolic parameters in NP#1

		NP#1	NP#2
		**MOLECULAR VARIANTS**	
***ATP7A* genetic variant**		c.3556delG, p.[Glu1186Serfs*3]	c.2626+1G>A
***ATP7A* expression (mRNA)**		Very low	Very low
**ATP7A protein**		Undetectable	Undetectable
		**PATIENT FEATURES**	
**Age**		20 months old (full-term)	4 months old (2 months age corrected for prematurity)
**Cu-His treatment**		250 µg/24 hours SC	250 µg/12 hours SC
**Clinical status**		Hypotonic; severe developmental delay	Asymptomatic
**Seizures; EEG**		Absent; normal	Absent; normal
**Brain MRI**		Vascular tortuosity, slight delay in myelination pattern, slight brain atrophy [14-month-old]	Vascular tortuosity, no brain atrophy, regular myelination pattern [2 months corrected age]
**Bladder diverticulum**		Present	Absent
**Pulmonary CT scan**		Bullous emphysema	ND
**Hair microscopy**		#Pili torti, trichorrhexis nodosa,and lack of melanin granules in hair structures (frontal + occipital regions) [23-month-old]	Pili torti, trichorrhexis nodosa, lack of melanin granules in hair structures (frontal + occipital regions) [7-month-old]
		**BIOCHEMICAL PARAMETERS**	
	**Reference values**		
Blood			
**Copper**	620 – 1544 µg/L	726	1245
**Ceruloplasmin**	200 – 470 mg/L	236	206
**Dopamine**	10 – 150 ng/L	31	ND
**Norepinephrine**	300 – 650 ng/L	73	ND
**Epinephrine**	20 – 60 ng/L	20	ND
**Urea**	5 – 25 mg/L	27	10
**Creatinine**	<0.50 mg/L	0.50	0.28
**Cystatin C**	0.60 – 1.20 mg/L	1.53	1.56
Urine			
**β_2-microglobulin**	<0.3 µg/L	11.6	18.6

**CEREBROSPINAL FLUID (CSF) METABOLITE LEVELS IN NP#1**				
**Biochemical metabolites**	**ES-Cu treatment week 17**	**ES-Cu treatment week 69**	**Units**	**References values**
Copper	5	2.1	µg/L	4.2 – 19
3-ortho-methyldopa	26	24	nmol/L	40 – 50
3-methoxy-4-hydroxyphenylglycol (MHPG)	38	28	nmol/L	20 – 80
5-hydroxytryptophan (5-HTP)	12.7	15.5	nmol/L	0 – 26
5-hydroxyindolaceitic acid (5-HIAA)	209	160	nmol/L	170 – 490
Homovanillic acid (HVA)	486	471	nmol/L	344 – 906
HVA/5-HIAA ratio	2.33	2.94	nmol/L	1.5 – 3.5
HVA/MHPG ratio	13	16.82	nmol/L	5 - 30

Hair specimens were obtained after starting the ES-Cu treatment; ND: not done

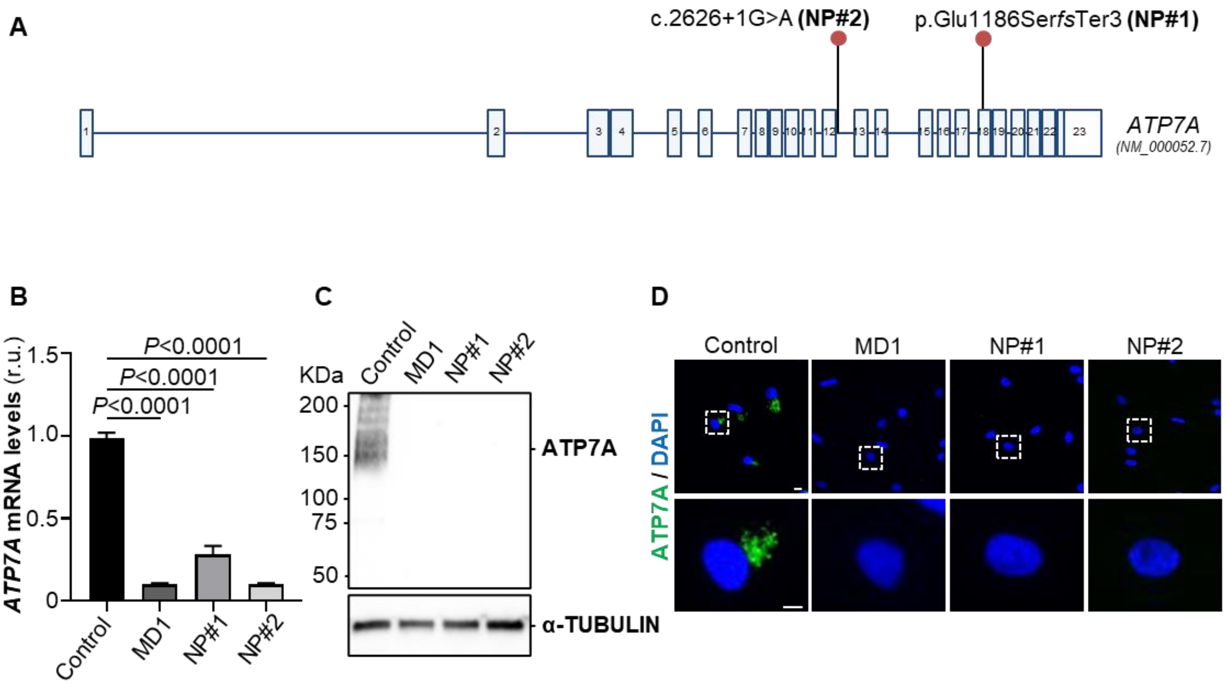

Supplemental Figure 1. Genetic and molecular characterization of *ATP7A* in NP#1 and NP#2. (A) A schematic of *ATP7A* gene with the location of pathogenic variants in NP#1 and NP#2. **(B)** *ATP7A* expression analysis by RT-qPCR in fibroblasts from a healthy control, an unrelated Menkes disease patient (MD1), and NP#1 and NP#2. Data are expressed as relative means ± SD; One-way ANOVA followed by Tukey-Kramer post-hoc test. **(C)** Western blot analysis of ATP7A (163.4 kDa) in fibroblasts from healthy control, MD1, NP#1, and NP#2. α-Tubulin is used as a loading control. **(D)** Immunofluorescence of ATP7A (green) and nuclear staining with DAPI (blue) in fibroblasts from healthy control, MD1, NP#1, and NP#2. Scale bar 10 µm (upper panel) and scale bar 20 µm (lower panel). Abbreviations: r.u., relative units.

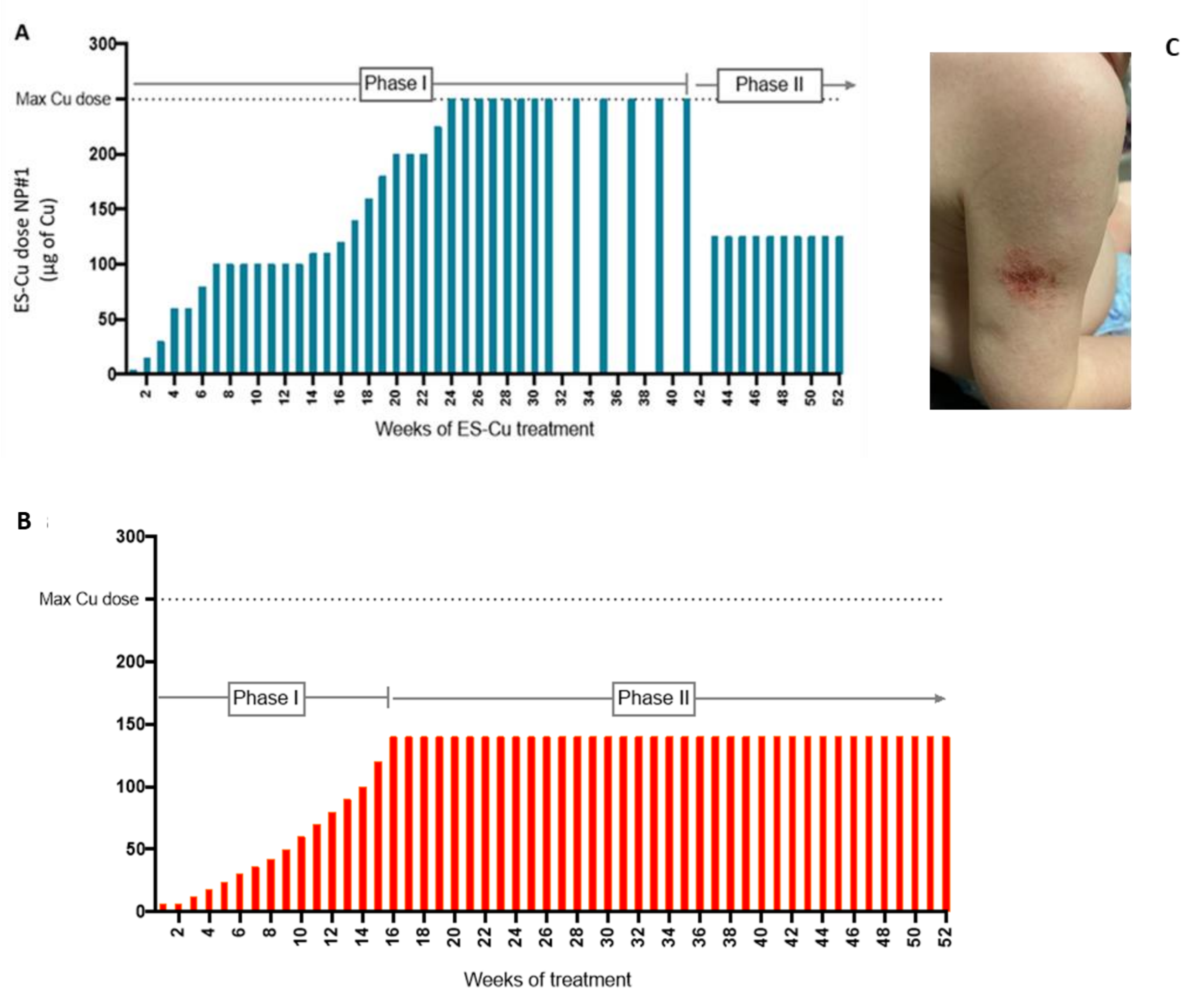

Supplemental Figure 2. **Dose escalation and skin reaction. (A and B)** Dose escalation in NP#1 **(A)** and NP#2 **(B)**. Due to ES-Cu injection site reactions in the arm during week 31 of treatment in NP#1 **(C)**, the 250 µg doses were administered every two weeks and subsequently adjusted to 125 µg per week.

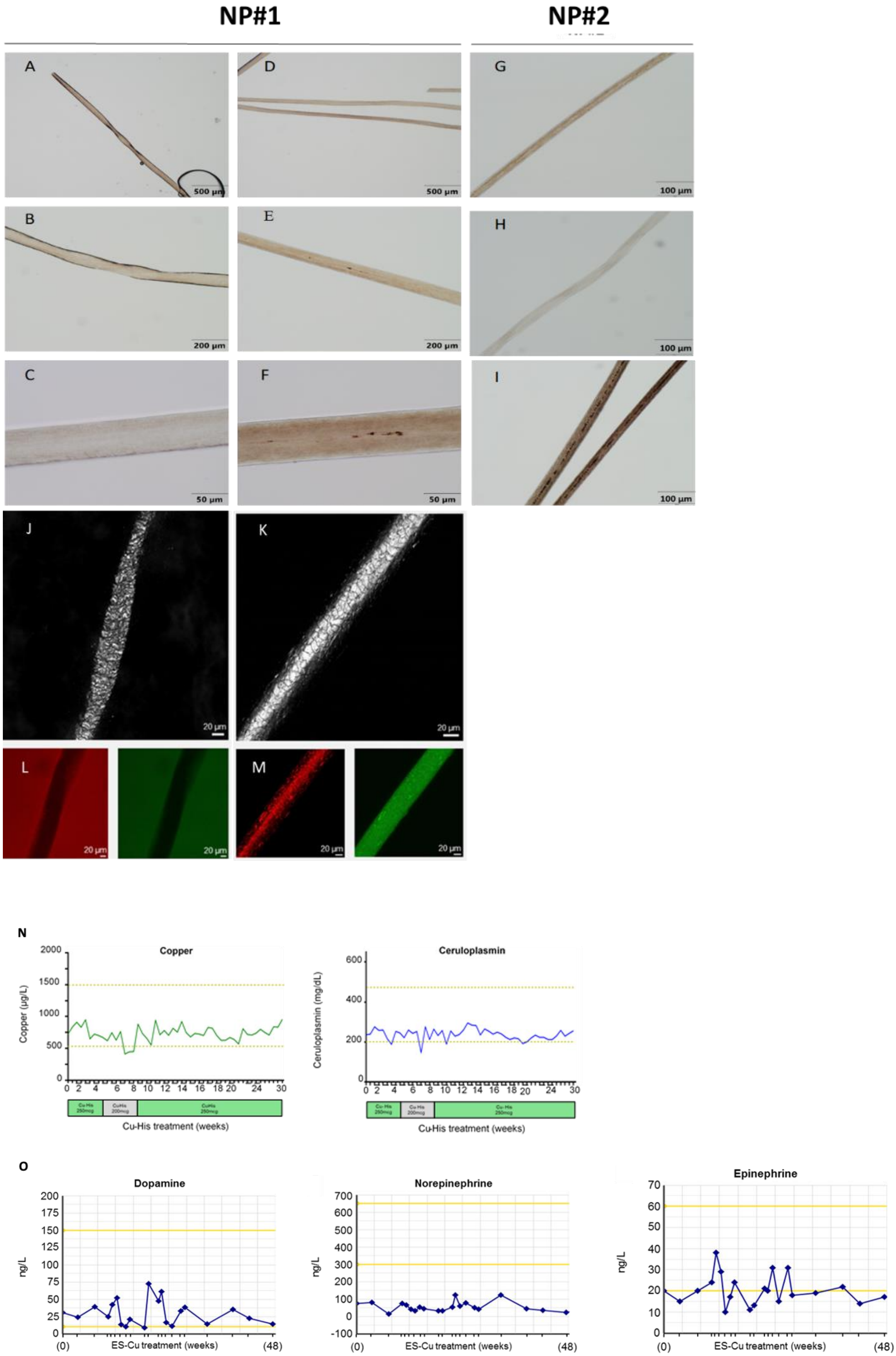
NP#1
NP#2
A
500 μm
D
500 μm
G
100 μm
B
200 μm
E
200 μm
H
100 μm
C
50 μm
F
50 μm
I
100 μm
J
20 μm
K
20 μm
L
20 μm
20 μm
M
20 μm
20 μm
N
Copper
Copper (μg/L)
Cu-His treatment (weeks)
Ceruloplasmin
Ceruloplasmin (mg/dL)
Cu-His treatment (weeks)
O
Dopamine
ng/L
(0)
ES-Cu treatment (weeks)
(48)
Norepinephrine
ng/L
(0)
ES-Cu treatment (weeks)
(48)
Epinephrine
ng/L
(0)
ES-Cu treatment (weeks)
(48)

Supplemental Figure 3. Hair and biomarker analysis. (A-I) Light microscopy of hairs from NP#1 and NP#2. For NP#1, after 3 months of treatment, longitudinally oriented hair appeared, and cases of pili torti and trichorrhexis nodosa were observed **(A, B)**. The hair color exhibited a significant reduction in melanin, resulting in a whitish appearance **(C)**. After 19 months of treatment, there were no signs of pili torti **(D, E)**, and the hair displayed a brownish color with central melanin granules **(F)**. For NP#2, at 1 month of age (-1 month corrected age), there were no observable central melanin granules or pili torti **(G)**. By 16 months of corrected age, isolated hairs exhibited alterations in the shaft resembling incomplete pili torti **(H)**. By 23 months of corrected age, no neurotrichosis changes were noted, and most hairs exhibited central melanin granules **(I)**. **(J-M) Confocal microscopy of hairs from NP#1:** The 3D projection of the reflection mode (grey) showing the hair shaft after 3 **(J)** and 19 **(K)** months of ES-Cu treatment. At 19 months of treatment, the cuticle, composed of layers of superimposed cells in the form of imbricated, overlapping flat scales, typical of healthy hair, can be observed. **(L)** Absence of fluorescence in red and green range at three months of treatment. **(M)** At 19 months of treatment, the hair exhibits autofluorescence in these emission ranges due to the presence of intact keratin. **(N-O) Monitoring blood metabolic biomarkers in NP#1**. After starting ES-Cu, Cu and ceruloplasmin were measured for 30 months **(N),** and catecholamines were measured for 48 months **(O)**.
