# Supplementary figures and images for: Elesclomol-copper therapy improves neurodevelopment in two children with Menkes disease
Source: J Clin Invest. 2025 Jul 29;135(19):e193107. doi: 10.1172/JCI193107 (PMC12483558; doi:10.1172/JCI193107)

**Overlay: Chemi+membrane**

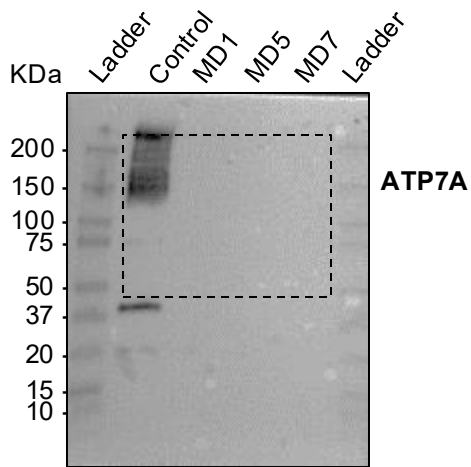

**Re-hybridized with  $\alpha$ -TUBULIN: Chemi+membrane**

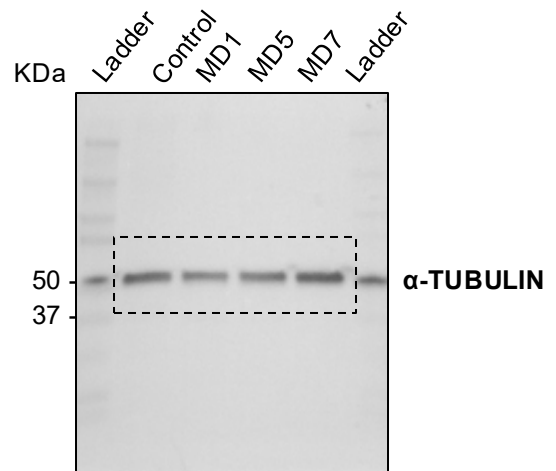

Supplement: Unedited blot and gel images [file jci-135-193107-s100.pdf]
